# Aberrant Left Testicular Artery Originating from the Inferior Mesenteric Artery Identified on Angiography in a Patient with Gastrointestinal Bleeding: Case Report

**DOI:** 10.3390/reports8020086

**Published:** 2025-06-03

**Authors:** Sarah M. Taheri, Connor S. Centner, Rahim H. Shalash, Touqeer Sulehria, Nana Ohene Baah

**Affiliations:** 1 School of Medicine, University of Louisville, Louisville, KY 40204, USA; 2 Division of Interventional Radiology, Department of Radiology, University of Louisville Hospital, Louisville, KY 40204, USA

**Keywords:** anatomic variation, angiography, embolization, therapeutic, mesenteric artery, inferior, mesenteric artery, superior

## Abstract

**Background and Clinical Significance:** This case presents a rare variation in mesenteric and pelvic vasculature that holds relevance for endovascular procedures. Limited published cases of the testicular artery arising off the inferior mesenteric artery exist in the literature and play an important role in clinical outcomes. **Case Presentation:** An 89-year-old male presented with gastrointestinal bleeding from diverticulosis. During an arteriogram to locate and assess sigmoid arteries for embolization, an unusual anatomical variant of the left testicular artery was discovered. Typically, the left testicular artery originates from the abdominal aorta below the renal arteries. However, in this patient, the left testicular artery was found to directly branch off the inferior mesenteric artery, while the superior rectal artery was absent from the inferior mesenteric artery. **Conclusions:** Awareness of such vascular variations is essential for interventionalists to optimize procedural success and minimize complications. Recognizing potential vascular anomalies, such as those presented in this case, is essential for effective pre-procedural planning and intraoperative management to improve patient outcomes.

## 1. Introduction and Clinical Significance

Embolization is a commonly performed procedure by interventional radiologists to control or prevent bleeding by selectively reducing blood flow to targeted regions. While the left testicular artery (LTA) typically arises from the abdominal aorta, significant variants in the anatomical presentation can occur with high or low origins off the abdominal aorta or branching off the renal artery being the most frequent sources [1]. A 2018 meta-analysis investigating documented variations in the testicular artery identified 1007 initial records, with 477 satisfying the inclusion criteria. Among these, 62 cases described variations involving an abnormal origin site of the LTA [1]. Most of these anomalies reported the LTA arising from the renal artery, but none cited the LTA originating from the inferior mesenteric artery (IMA). Here, we report a case of multiple vascular abnormalities where the LTA originates directly from the IMA while the superior rectal artery (SRA) is absent from the IMA. Despite these variations, the embolization of the sigmoid artery was successfully performed to treat a recurrent diverticular gastrointestinal bleed (GIB) with no major complications.

The clinical significance of this aberrant testicular artery anatomy is far-reaching within interventional radiology. Common radiologic interventions involving the IMA include embolization, stenting, angioplasty, and diagnostic angiography. Embolization of the IMA poses a particular risk in the presence of this anatomical LTA variant. The IMA is commonly embolized during endovascular aortic repair for treatment of abdominal aortic aneurysm to prevent type II endoleak [2]. As the primary blood supply to the left testes, disruption of blood flow to the LTA from embolization of the parent artery may result in significant infarction. Testicular infarction of any cause can result in possible necrosis or loss of the testicle [3]. Vigilance with respect to all branches, especially those that are atypical, of the IMA during these procedures, as well as increased awareness with respect to the potential for the LTA specifically to originate from the IMA, will be of the utmost importance clinically to prevent potential poor outcomes in patients presenting with this anatomy.

## 2. Case Presentation

An 89-year-old male with a history of diverticulosis with sigmoid GIB presented to interventional radiology for assessment and treatment. To gain right common femoral artery access, a 21-gauge micro-puncture needle was used. Subsequently, a 5 French catheter was placed, and an abdominal aortogram was performed.

**Figure 1:**
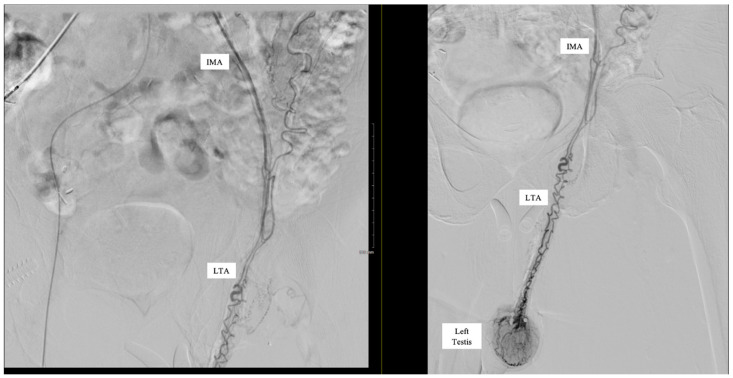
Inferior mesenteric artery (IMA) angiogram demonstrating accessory artery supplying left testis.

**Figure 2:**
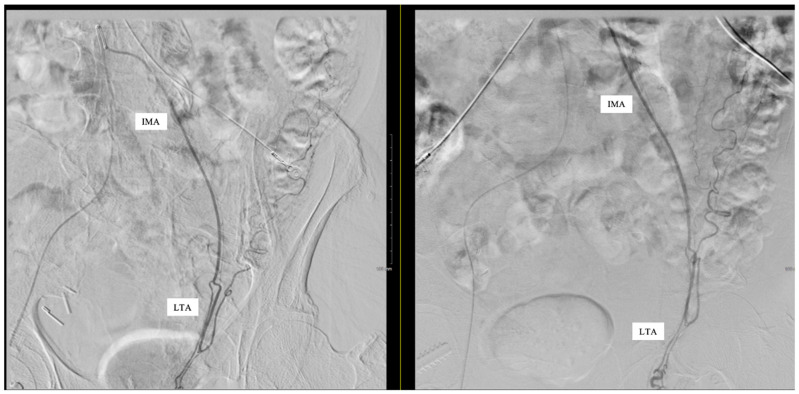
Left testicular artery (LTA) originating from the inferior mesenteric artery (IMA). No superior rectal branch was present.

Subselective catheterization of the IMA was performed, and an angiogram demonstrated an aberrant LTA, shown in Figure 1. The sigmoidal branch was also identified; however, the superior rectal branch was absent from the IMA, as shown in Figure 2. Additionally, no active extravasation was identified. Selective catheterization of the artery supplying the sigmoidal distribution was performed, and Gelfoam embolization was performed prophylactically due to previous bleeding episodes involving the sigmoid distribution on the prior CT angiography (CTA). Postembolization arteriograms demonstrated sufficient occlusion.

Following embolization, catheterization and subsequent angiograms of regional arteries that may have been impacted revealed no evidence of active extravasation or bleeding. Subsequently, all wires and catheters were removed, a vascular closure device was deployed, and the patient was discharged in stable condition.

## 3. Discussion

The left and right testicular arteries are typically paired, originating directly from the abdominal aorta at the level of approximately L1–L3 [4]. However, anatomic variation is commonly seen. Embryologically, the testicular arteries arise from the lateral mesonephric arteries in the fetus. The lateral mesonephric arteries are divided into cranial, middle, and caudal groups, with the testicular arteries typically arising from the caudal group [5]. Abnormal regression of these mesonephric arteries can lead to differing origins and presentations of the testicular arteries and account for much of the anatomical variation commonly seen [6]. The LTA is more commonly seen with anatomical variation when compared with the right [1]. The most common testicular artery variations are originating higher or lower of the abdominal aorta, branching off the renal artery, or having a double LTA [1]. Less commonly seen, testicular arteries may share a common trunk of the abdominal aorta or originate as branches from the subcostal artery, middle suprarenal artery, thoracic aorta, common iliac artery, external iliac artery, or from the inferior epigastric artery [1]. Arching of the testicular arteries or complete absence of a testicular artery can also be seen [1]. In contrast, the LTA branching from the IMA is considered an extremely rare anatomic variant, with a case report by Messanna et al. [7] being the only known documented occurrence of this variation in the LTA site of origin. Other meta-analyses examining thousands of records on anatomical variations involving the testicular arteries failed to identify any documented instances of this vascular anomaly [1].

The IMA is a major unpaired branch of the abdominal aorta and, along with the celiac trunk and superior mesenteric artery, is one of three primary splanchnic arteries. The IMA has three primary branches: the left colic (LCA), the sigmoid, and the SRA. It supplies blood to the distal third of the transverse colon, the descending colon, the sigmoid colon, and the superior portion of the rectum, which together embryologically comprise the hindgut. The IMA originates from the abdominal aorta at level L3 and is considered to be relatively regular in its anatomy, such that major anatomical variation is uncommon [8]. There is understood to be some variation in regard to whether the three primary arteries of the IMA arise from three distinct branches or whether the sigmoid artery shares a branch with the LCA or the SRA [9]; however, additional aberrant arteries branching from the IMA outside of these primary arteries, as was seen in our case, are uncommon.

An LTA arising directly from the IMA has been rarely reported [7]. The testicular artery has been reported to arise from the abdominal aorta at the level of the IMA in other cases [10], but this is distinct from the LTA arising directly from the IMA. In our case, we also found that the SRA was not present as a branch of the IMA, which was consistent with the findings of Messanna et al. [7], although they were later able to locate the SRA as a branch of the median sacral artery for embolization for treatment of hemorrhoidal disease. In our case, the patient’s sigmoid artery was targeted for embolization for treatment of GIB secondary to diverticulosis. It is imperative for interventional radiologists to be aware of atypical anatomical presentations that may be encountered to mitigate unintended outcomes.

This case emphasizes the importance of having awareness of vascular variations, as well as both pre-procedural planning and intra-procedural vigilance in endovascular procedures. Extremely rare anomalies, such as the LTA originating from the IMA presented in this case, increase the risk during procedures of inadvertent negative impacts on testicular blood flow. Acute cessation of blood supply to the testes has been shown to be catastrophic, potentially resulting in testicular ischemia or infarction [4]. Appropriate evaluation of pelvic vasculature via approaches such as angiographic evaluation and awareness of anatomical variations such as the anomalies presented in this case have been strongly connected to the success of embolization procedures in this region [11].

More broadly, this case highlights the value of pre-procedural planning to mitigate intraprocedural complications. Approaches such as cross-sectional CT angiograms may help predict variations in vascular anatomy that can guide procedural approaches and reduce the risk of complications [12]. In fact, artificial intelligence could enhance pre-procedural planning by identifying and labeling abnormal vasculature. This ultimately could reduce interpretation time and improve accuracy in complex cases by helping proceduralists navigate challenging vasculature and avoid off-targeted embolization.

## 4. Conclusions

An aberrant LTA originating from the IMA was unexpectedly encountered when performing an arteriogram and embolization of the sigmoid artery to treat GIB. Interventionalists should be aware of the atypical anatomy of the LTA and potential abnormal anatomical origin from the IMA when performing embolization procedures to ensure effective treatment and minimize unintended procedural challenges. Preprocedural and intraprocedural imaging can mitigate off-target effects. These techniques can be further supplemented by artificial intelligence.

## Data Availability

The original contributions presented in this study are included in the article. Further inquiries can be directed to the corresponding author.

## Abbreviations

The following abbreviations are used in this manuscript:

LTA	Left Testicular Artery
IMA	Inferior Mesenteric Artery
SRA	Superior Rectal Artery
GIB	Gastrointestinal Bleed
CTA	Computed Tomography Angiography
LCA	Left Colic Artery
